# A Semantic-Based Gas Source Localization with a Mobile Robot Combining Vision and Chemical Sensing

**DOI:** 10.3390/s18124174

**Published:** 2018-11-28

**Authors:** Javier Monroy, Jose-Raul Ruiz-Sarmiento, Francisco-Angel Moreno, Francisco Melendez-Fernandez, Cipriano Galindo, Javier Gonzalez-Jimenez

**Affiliations:** Machine Perception and Intelligent Robotics group (MAPIR), Department of System Engineering and Automation, Biomedical Research Institute of Malaga (IBIMA), University of Malaga, 29071 Málaga, Spain; jotaraul@uma.es (J.-R.R.-S.); famoreno@uma.es (F.-A.M.); fco.melendez@uma.es (F.M.-F.); cga@uma.es (C.G.); javiergonzalez@uma.es (J.G.-J.)

**Keywords:** e-nose, electronic nose, gas sensor, mobile robot, gas source localization, semantics, uncertainty, sensor fusion, object recognition, MDP, Markov decision process, gas classification, Bayes, uncertainty propagation, Mobile Robot Olfaction, chemical sensors, Artificial Intelligence, Machine Learning

## Abstract

This paper addresses the localization of a gas emission source within a real-world human environment with a mobile robot. Our approach is based on an efficient and coherent system that fuses different sensor modalities (i.e., vision and chemical sensing) to exploit, for the first time, the semantic relationships among the detected gases and the objects visually recognized in the environment. This novel approach allows the robot to focus the search on a finite set of potential gas source candidates (dynamically updated as the robot operates), while accounting for the non-negligible uncertainties in the object recognition and gas classification tasks involved in the process. This approach is particularly interesting for structured indoor environments containing multiple obstacles and objects, enabling the inference of the relations between objects and between objects and gases. A probabilistic Bayesian framework is proposed to handle all these uncertainties and semantic relations, providing an ordered list of candidates to be the source. This candidate list is updated dynamically upon new sensor measurements to account for objects not previously considered in the search process. The exploitation of such probabilities together with information such as the locations of the objects, or the time needed to validate whether a given candidate is truly releasing gases, is delegated to a path planning algorithm based on Markov decision processes to minimize the search time. The system was tested in an office-like scenario, both with simulated and real experiments, to enable the comparison of different path planning strategies and to validate its efficiency under real-world conditions.

## 1. Introduction

Mobile robots operating in human environments such as offices, hospitals, or factories, benefit from the fusion of different sensing modalities to efficiently accomplish tasks that are hard or even unfeasible to address if only one sensor is employed [1]. In this work, we focus on two of these modalities, namely vision and olfaction, and study their application to a challenging problem: the localization of gas emission sources within real-world indoor environments, commonly referred as gas source localization (GSL) [2]. An efficient solution to this problem would permit a mobile robot to act fast and according to the situation, for instance by alerting a human (e.g., notifying the presence of smoke from the oven, or a harmful leak from a particular pipeline within a factory) or by suggesting different actions to be carried out (e.g., preventive maintenance on some machinery that starts to release a burnt smell or replacing the pet sandbox). In this context, *vision* is considered as the perception of the robot workspace through a camera capturing light intensity [3], while *olfaction* is the sensing of volatile chemical substances by an electronic nose (e-nose) [4].

Traditionally, GSL has been addressed by mimicking animal behaviors through bio-inspired algorithms, assuming the existence of a downwind gas plume (i.e., plume tracking) [5,6], or by exploiting information sources such as gas dispersion models or windflow data [7,8]. However, most of these methods are prone to fail in real-world environments due to the important assumptions they rely on, namely the existence of a well formed downwind gas plume, the predominance of laminar and uniform windflows, or the absence of obstacles in the environment that can interfere with the gas dispersion.

Tackling the GSL problem without relying on these assumptions is not a simple task, particularly when the only data sources are gas and windflow observations. Thus, it seems a natural approach to fuse new information sources such as vision to decrease the complexity of the search process by narrowing the search space to a set of potential candidates to be the gas source, to which the recognition of objects through computer vision techniques can contribute to a large extend. For example, if the e-nose detects an abnormal concentration of a gas that is classified as rotten food, an object recognized as a bin is a potential candidate, while a heater is not. While not novel, this approach has only been superficially explored in the literature with very simple problem domains, where the robot exploited prior knowledge about the source physical characteristics to reduce the locations to search [9]. Moreover, it is still missing a formal way to define and exploit the relationships among gases and objects—i.e., their semantics—which could assist the GSL process in a more flexible way.

In this work, we contribute with a novel gas source localization system that pursues both efficiency by exploiting the semantics between the detected gas and the objects in the environment, and coherence through the consideration of the uncertainty in the identification of gases and objects. The latter is fundamental since neither gas classification nor object visual recognition is exempt from uncertainty sources such as the cross-sensitivity of gas sensors, the light conditions, or the models used to identify them. In this way, the system uses an ontology [10] to encode the semantics involved in the GSL problem, including information relating gases and objects (e.g., that heaters can give off smoke or gas smells), as well as information gathered from the environment (e.g., that a heater was recognized at a certain location).

This semantic is leveraged to provide valuable prior information, namely: the categories of objects that can give off the target smell, the objects/instances of these categories that have been recognized so far in the environment, and their locations. The system assumes that the robot knows the environment and that different objects have been detected and categorized in advance. Notice that this does not imply that the robot must have knowledge of all the objects in the environment, which would be unrealistic, but as the search progresses new objects can be added as source-candidates by either visual or chemical observations (see Section 3.1 and Section 3.2). Both semantics and classification results feed a probabilistic Bayesian framework, which propagates the related uncertainties, and assigns to each object a probability of being the gas source. Finally, a path planning algorithm based on Markov decision processes (MDP) merges these probabilities with the distances from the current robot location to the objects, to produce a plan that minimizes the search time.

We demonstrated the suitability of our search strategy by simulating a 3D office-like environment with multiple objects. We used of computational fluid dynamic (CFD) tools and a gas dispersion simulator (GADEN [11]) to reproduce realistic gas releases in the environment where a mobile robot must locate the object releasing the gas. Thanks to the repeatability introduced by simulating the gas dispersal, a comparison between different path planning strategies is provided. Then, a real experiment was conducted to validate our approach under real-world conditions, providing a trace of how the different probabilities involved in the search vary over time. Furthermore, we emphasize the dynamic nature of our approach by illustrating the case where the gas-source is not within the list of candidates, being necessary the detection of previously not detected objects.

## 2. Related Work

Gas source localization strategies relying on a mobile robot to sample the gases dispersed in an environment can be classified into those designed to work under the presence of a chemical plume, also known as plume tracking strategies, and those that do not rely on the existence of a well formed, downwind plume. The former includes approaches that rely only on gas sensing (*chemotaxis*) [2], and those that fuse *chemotaxis* with *anemotaxis* [12], exploiting the strong directional cue that the wind flow direction brings. The main drawback of these strategies is the fact that under structured, turbulent environments (e.g., with presence of objects such as walls and furniture that break down the plume), a great difficulty exists in accurately determining the plume, and therefore real experiments commonly fail to locate the source [13].

From the wide range of GSL methods that do not depend on a well formed plume, in this paper, we focus on two particular approaches: those that build upon vision systems to boost the search efficiency, and works that consider uncertainty during the search process. Vision-based strategies enable robots to identify potential candidates from a distance, thus dramatically diminishing the effective search space and greatly enhancing the ability to locate an odor source in a short time. However, and despite this notable advantage, only very basic algorithms have been proposed in the literature, most of them relying on strong assumptions about the gas-source shape or color for the visual detection of candidates [9,14]. An exception is the work proposed by Loutfi et al.  [15], who proposed a symbolic reasoning technique for fusing vision and olfaction. However, they focused on object recognition, where gas sensing is only employed for object disambiguation, not to locate the source releasing the volatiles.

With regard to GSL approaches considering some type of uncertainty in the search process, we can highlight strategies such as infotaxis [7], a gradient-free method that exploits the expected entropy of future samples to guide the robot search towards the gas source; the work presented in [16], where the authors proposed a probabilistic approach using partial differential equations to model the diffusion of gases in the environment and infer the location of one or multiple gas-sources; or the increasingly common approaches based on particle filters (e.g., [8,17]). However, most of these approaches have not yet been proved to work under the challenging conditions imposed by real-world human environments (i.e., with high turbulence, non-uniform wind fields, and the presence of obstacles), which is the focus of this work.

Those works addressing the problem of source term estimation (STE) where the goal is not just to locate the gas source but to estimate different parameters related to the gas dispersion deserve special mention. Common parameters include the location of the gas source, the emission rate, the wind conditions in the environment, or the diffusive constant. Inferring all these parameters is, in comparison with GSL, a more complex and challenging problem that often requires taking assumptions about some of these parameters. STE based approaches are common in the field of atmospheric dispersion [18,19] where assuming parametric dispersion models or uniform windflow conditions are feasible practices. In the mobile robotics community, only initial approaches to solve the STE problem have been proposed for the case of small indoor scenarios ([20,21]), considering controlled or semi-controlled experimental settings.

Finally, it is worth mentioning strategies based on gas distribution mapping, which do not rely on the presence of a gas plume or on strong assumptions about the environmental conditions, and are able to suggest likely locations of the gas source [22,23,24]. Their main drawback resides in the time necessary to inspect the entire environment and their bad scalability as the environment enlarges.

## 3. System Description

**Figure 1:**
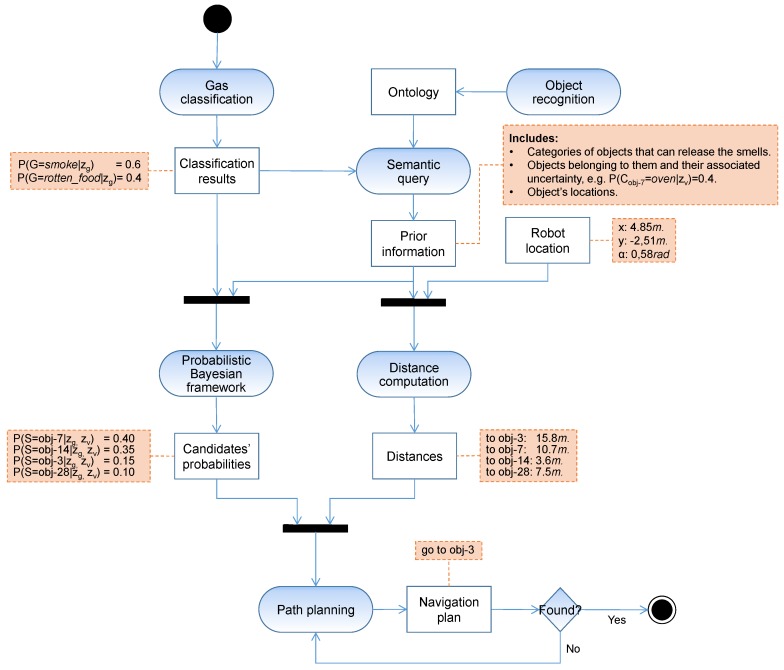
Activity graph (employing the unified modeling language (UML)) defining the workflow of the proposed system for semantic gas source localization.

The objective pursued in this work was to minimize the time employed in locating a gas emission source within a real-world human environment, given a mobile robot equipped with an e-nose able to measure and classify different chemical volatiles. Figure 1 illustrates an overview of the presented semantic gas source localization system (SGSL), including the handled data and the processes involved in it. Its execution is triggered with the detection of an abnormal gas concentration by the e-nose mounted on the robot, which, in turn, leads to its classification obtaining a list of gas classes and their respective beliefs (see Section 3.1).

Given the challenging conditions of real-world environments, particularly with regard to the chaotic gas dispersion and the impossibility to assume uniform wind flows, we propose in this work to fuse vision and chemical sensing instead of uniquely relying on the e-nose observations. By making use of the noteworthy advances in computer vision, we employed an object recognition system (Section 3.2) to detect and classify the objects the robot encounters along its path.

With this valuable information, the system narrows the locations where to search by considering as potential candidates all the objects detected that can give off those smells. This is done by performing a semantic query to an ontology (see Section 3.3). Taking into consideration the uncertainty related to the object recognition and gas classification processes, we propose to employ a probabilistic Bayesian framework to propagate such uncertainties and produce an ordered list with the most promising candidates to be the gas source (see Section 3.4) This set of candidate objects is dynamically updated as the robot operates, enabling the inclusion of new candidates not previously considered in the search (Section 3.5), making the proposed system more flexible and robust.

Assuming at this point that a set of objects have been previously recognized (notice that this is not a strong assumption, since for the case of having an empty set of candidates, the robot only has to trigger a *search-for-objects* behavior, as described in Section 3.5), our problem can be formally defined as:

(1) $$\underset{s_{s}}{arg min}\;\left\{t_{s}\left(s_{s}\right)|t_{s}\left(s_{s}\right)=\frac{d(s_{s})}{v}+n\left(s_{s}\right)\cdot t_{v}\right\},$$

where the argument $s_{s}$ refers to the search strategy, $t_{s}\left(s_{s}\right)$ represents the global search time reported by such strategy, *d* is the travelled distance by the mobile robot, *v* is the average robot speed, *n* accounts for the number of objects inspected before detecting the gas source (i.e., failed attempts), and $t_{v}$ is the validation time or time employed in ascertaining whether an object is releasing gases. Notice that the validation or gas source declaration, as it is commonly referred in the literature [25,26,27], can be considered a sub-task of the general gas source localization problem, determining the certainty that a gas source is in the immediate vicinity of the robot. Due to the turbulent character of gas transport in natural indoor environments, this declaration is by itself a very challenging problem. In this work, we do not address this sub-task, and only consider a very simple validation scheme where we assume the source has been found as long as the robot visits the object for inspection.

To properly describe the proposed approach from a probabilistic stance, we consider the following random variables:

- $z_{g}\in R^{l}$ is a certain gas measurement from the e-nose characterized through a vector of *l* features.
- $z_{v}\in R^{m}$ is an observation of an object from the camera characterized by *m* features.
- $G=\{G_{i},\;i=1:N_{G}\}$ models the gas class and takes values on the set of $N_{G}$ possible gases.
- $C=\{C_{i},\;i=1:N_{C}\}$ stands for the category of a candidate object, assigning to it a value from the set of $N_{C}$ categories.
- $S=\{o_{i},\;i=1:N_{O}\}$ stands for the gas source, taking values on the set of $N_{O}$ objects perceived so far in the environment.

Furthermore, we relied on the following two assumptions: (i) there is only one gas source emitting the target gas, that is, we account for the likely possibility that other gases may be present in the environment (treating them as interference gases), but the search will stop as soon as one candidate is validated as the source; and (ii) the gas source is an object that can be detected by the visual recognition system. The latter simplifies the problem by not considering the case where the gas source is undetectable by the robot, which will require fusing the current approach with other search strategies not relying on object recognition (not considered in this work).

Finally, and without losing the focus on minimizing the global search time $t_{s}$, a MDP-based path planning algorithm is introduced to consider the travelling cost to visit the candidates (see Section 3.6) and the time required for validating if the candidate at hand is the gas source or not. The next sections describe the system components in more detail.

### 3.1. Gas Detection and Classification

In this work, we assumed that a mobile robot deployed in a human environment is equipped with an e-nose that is sampling the environment on a regular basis. This implies that, while the robot is performing its duty tasks (e.g., patrolling, assistance, cleaning, etc.), it is also monitoring the gases present in the air. When an abnormal gas concentration level is detected, that is, when the gas concentration observed exceeds a predetermined threshold, the SGSL system is triggered.

At this point, a classification task is carried out to determine which objects in the environment are susceptible of releasing the observed gas. As previously mentioned, we accounted for the uncertainty in this process by considering probabilistic classifiers [28,29], that is, the output is not a unique class label, but a set of posterior probabilities representing the belief of the gas observation to belong to each considered gas-class: $P(G_{i}|z_{g})$. Any gas classifier giving as output a probability distribution over the set of classes can be employed, e.g., Support Vector Machines, Naive Bayes (the one considered in this work), Decision Trees, etc.

### 3.2. Object Detection and Recognition

The detection and categorization of objects in the environment is a key component of the system, necessary to populate the set of candidates to be the gas source. For that, similar to the classification of gasses, any object recognition system defining $P(C_{j}|z_{v})$ can be used, i.e., providing a probability distribution over the results that models the uncertainty inherent to the recognition process [30,31]. For the real experiments in this work, we relied on a state-of-the-art Convolutional Neuronal Network (CNN), namely You Only Look Once v3 (YOLOv3) [32], given its high recognition success. In the case of our robot, and as in most robotic platforms, the available computational resources are limited, so we employed its tiny version, a reduced model for constrained environments that exhibits a balanced performance. This network provides bounding boxes containing the objects detected in the image and a belief about their classification as belonging to certain categories. For a given object, the sum of these beliefs can be equal to or less than 1, thus, to build a categorization probability, we considered the remaining amount as the probability of belonging to an arbitrary object category that does not release any gas. For example, if the CNN recognizes an object as a chair with a belief of 0.8, then $P(C_{j}=Chair|z_{v})=0.8$ and $P(C_{j}=Arbitrary|z_{v})=0.2$, being zero the probability for the remaining categories.

Once an object is detected, it has to be located in the environment, i.e., their pose must be expressed in global coordinates. For that, we relied on a RGB-D camera that yields both intensity and depth images: YOLO is executed over the intensity ones to detect objects, while their bounding boxes are propagated to the depth images, permitting the objects in the local camera frame to be located. Given the extrinsic calibration parameters of the camera with respect to the robot frame [33], the object poses can be expressed in coordinates of the latter which is, at the same time, located in the global frame. Notice that this recognition system is continuously running on the robot, so new objects can be detected and recognized at any moment.

### 3.3. Ontologies and Semantic Queries

Once the detected gas is classified, e.g., the gas can be smoke with probability 0.7 or rotten food smell with 0.3, the goal is to search for the objects that can give off these smells. To obtain that information, a prerequisite is to know the categories of objects that can release them, i.e., the semantics among smells and object categories. Once such a knowledge is available, the search can focus on the instances of that categories in the robot workspace. In this work, we opted for using ontologies to codify this information in a principled way.

**Figure 2:**
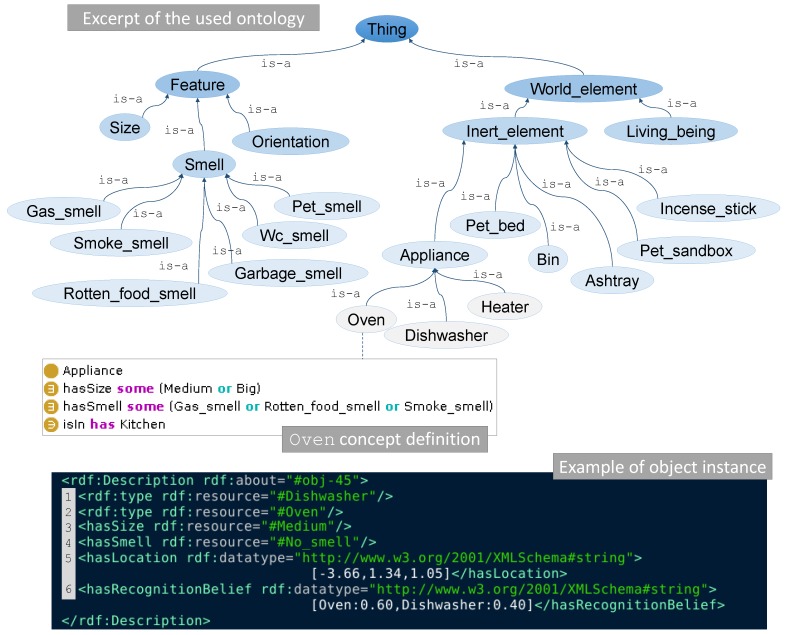
(**Top**) Excerpt of the ontology used in this work, whose hierarchy is built by means of is-a predicates, e.g., is-a(Smell,Feature). (**Middle**) Simplified definition of the concept Oven describing the ovens usual size (medium or big), the smells that they can release (gas, rotten food or smoke) and their typical location (kitchens). (**Bottom**) Example of an object instance (obj-45) expressed in OWL. From the definition, we can retrieve that: the object could be an oven with belief 0.60 or a dishwasher with 0.40 (line 6, hasRecognitionBelief predicate), so both categories are added as types (Lines 1–2), it has a medium size (hasSize predicate, Line 3), it is not releasing any smell (hasSmell, Line 4), and its location in global coordinates is x=−3.66 m, y=1.34 m and θ=1.05 rad (hasLocation, Line 5).

An ontology can be defined as a formal and structured representation of the relevant knowledge about a domain of discourse [10,34]. An ontology O codifies a set of predicates $O=\{P_{1},\ldots ,P_{n}\}$ which refers to concepts in the domain, for example different types of smells, objects categories, etc. Ontologies are represented as hierarchies, which are modeled employing the is-a(A,B) predicates: is-a(Smoke_smell,Smell) tells us that Smoke is a concept that can be abstracted in the Smell one. Instances of these concepts can be also introduced through the instance-of(a,B) predicate, e.g., instance-of(obj-1,Oven) sets that the object identified as obj-1 is an individual of the Oven concept. Other predicates can be used to further describe concepts or individuals, such as hasSize(obj-1,Medium) that characterizes the object obj-1 has having a Medium size. Figure 2 shows an excerpt of the ontology used in this work, as well as the description of the concept Oven and an individual (obj-45), where the predicates P are expressed in the web ontology language (OWL).

Once defined, the ontology can be used for checking its consistency or coherence, inferring new information, or querying for pieces of it. In this work, we are interested in the latter, since given the smells we are looking for, it enables us to perform semantic queries for retrieving, first, the categories of objects that can emit them, and then, the instances of these categories previously detected in the robot workspace. These queries can be efficiently carried out using *SPARQL*. To complete the prior information provided by the ontology, it also yields the location of the candidate objects, which is needed to optimize the path for validating them (see Figure 1).

### 3.4. Fusing Information with the Bayesian Probabilistic Framework

Once the gas classification results and the information about the objects in the robot workspace are available, all this knowledge is fused by a Bayesian framework to consistently assign a probability of being the source to each candidate. That is, the probability of a certain candidate $o_{i}$ for being the gas source *S*, given a gas observation $z_{g}$ and the object visual observation $z_{v}$, is computed as:

(2) $$\begin{array}{c} P\left(S=o_{i}|z_{g},z_{v}\right)=\sum_{j=1}^{N_{C}}P\left(S=o_{i}|z_{g},z_{v},C_{j}\right)P\left(C_{j}|z_{v}\right)\\ P\left(S=o_{i}|z_{g},z_{v},C_{j}\right)=\sum_{k=1}^{N_{G}}P\left(S=o_{i}|C_{j},G_{k}\right)P\left(G_{k}|z_{g}\right) \end{array}$$

Thereby, each candidate probability is retrieved by a double marginalization: over the object categories in *C*, and over the gas classes in *G*. In this way, such probability is the result of the product of three conditional probability distributions. The first one, $P(C_{j}|z_{v})$, models the likelihood of the object $o_{i}$ to belong to a certain category $C_{j}$ (e.g., dishwasher, oven, bin, pet sandbox, etc.) conditioned on the observation from the camera $z_{v}$. Note that we are assuming that $C_{j}⫫z_{g}|z_{v}$, i.e., the object category is independent of the gas observation given the visual observation of the object. This probability is defined by the uncertain results of the object recognition process (e.g., $o_{i}$ can be an oven with belief 0.60, or a dishwasher with 0.40), which are codified into the ontology (recall Section 3.2 and Line 6 in the bottom part of Figure 2), and later retrieved by a semantic query to it.

The second distribution $P(S=o_{i}|C_{j},G_{k})$ represents the probability of $o_{i}$ being the source *S* conditioned on the source category $C_{j}$ and the type of gas $G_{k}$ being released. Again, we can safely assume that $S=o_{i}⫫z_{v},z_{g}|C_{j},G_{k}$. This probability is computed from the prior information (i.e., expert knowledge) encoded within the ontology about the compatibility of an object from category $C_{j}$ releasing a smell of class $G_{k}$. For example, according to the definition of the concept Oven in the middle part of Figure 2, ovens are compatible with (i.e., can release) gases of type rotten food or smoke. Therefore, only for those two smells the $P(S=o_{i}|C_{j},G_{k})$ should have a non-zero value, indicating that we do not consider the fact that ovens may release other gases. Naturally, the specific values assigned to each combination is dependent of the application domain. Table 2 illustrates an example of this expert knowledge in the form of conditional probabilities linking a set of object categories and gas classes for the experiments described in Section 5. For simplicity, we consider that $P\left(S=o_{i}|C_{j},G_{k}\right)=1/N_{p}$ if the object category can release such smell, and zero otherwise. $N_{p}$ stands for a normalization value codifying the total number of object categories that can release a specific gas, so it can be interpreted as a *normalized* indicator function.

Finally, $P(G_{k}|z_{g})$ models the probability of the detected gas being of class $G_{k}$ conditioned on the gas observation $z_{g}$, which corresponds with the output of the gas classifier (recall Section 3.1), safely assuming that $G_{k}⫫z_{v},C_{j}|z_{g}$. Given these three distributions, the computation of the probability of each candidate being the gas source can be accomplished efficiently (see Section 4).

### 3.5. Dynamic System Update

The proposed GSL system relies on a set of detected objects in the environment to estimate, for each one, their probability of being the gas source. Assuming that the gas source is within this set is unrealistic, therefore, in this section, we describe two strategies that have been implemented to dynamically account for objects not previously detected in the environment.

- **Passive Search:** The first and more straightforward strategy consists of continuously and permanently detecting and recognizing objects with the on-board perception system (i.e., a RGB-D camera) as the robot searches for the source. Since object recognition algorithms are computationally heavy, and assuming that the robot moves, on average, at low speeds, we can safely execute this task at a low rate (e.g., 1 Hz) maintaining a high detection rate.
- **Active Search:** The second strategy is triggered when the e-nose measures a concentration level of the target gas that exceed a configurable threshold. At this moment, the robot will hold the current source-search, and carry out an active exploration of the surroundings. The main difference with the passive search is that, in this mode, the robot movements are controlled to inspect the surroundings, while in the passive mode are not. In this work, we propose an inspection pattern described by the following sequence of movements:
  1. Perform a 360deg pure rotation.
  2. Randomly navigate to a location within a M×M square area centered on the location where the gas hit was observed.
  3. Repeat Steps 1 and 2 N times.
  The number of random navigations to carry out (N) as well as the size (M) of the area to search are parameters that must be set according to the workspace. For example, for the real experiments carried out in Section 5, we selected N=2 and M=4 m. Finally, to avoid repetitive calls to this active search behavior (e.g., in the surroundings of the gas source where high concentrations are expected to be measured), a timeout parameter $T_{o}$ is set that ensures a minimum time between two consecutive calls.

On the event of a new candidate detection (by either of the two object-search strategies described above), it is first checked with the ontology to discern whether this is a new object (in which case is added to the list of current candidates) or a new observation of an already detected object (nothing is done). Only under the case of an inclusion, the system needs to re-plan the current source-search by first updating the source probabilities of the remaining candidates, and then re-computing the associated utilities as described in the next section.

### 3.6. Path Planning with Markov Decision Processes

Once the probability of each object in the environment of being the gas source is computed, the robot must make a plan to visit and inspect them. For this step, we rely on a path planning module based on Markov decision processes (MDPs) [35]. MDPs have been successfully applied to estimate the global probabilities of success in navigation tasks within changing, uncertain environments [36]. An MDP can be defined by the tuple *(S,A,T,R,γ)* with *S* and *A* being finite sets of states and actions, *T: S × A→S’* the transition function that defines the probability of reaching the state *s’* if action *a* is executed from state *s*, *R: S × A→IR* the reward that the decision framework expects to receive when the state *s* is reached applying action *a*, and γ a time-discount constraint used to limit the evaluation horizon. In this way, we build our MDP model upon a topological map augmented with information about the traversability and execution-time of a given path. More concretely, we consider that:

- A state *s* is fully determined by a 2D robot location in the workspace (i.e., the (x,y) coordinates), a flag indicating if the current state is goal (i.e., the candidate is the gas source), and the list of objects not inspected yet.
- An action *a* represents the robot navigation from $o_{i}$ to $o_{j}$, and the validation of $o_{j}$ to be the source (true/false).
- The transition function *T* codifies the probability of an inspected object to be the gas source. Given that an action *a* involves inspecting a particular object location ($o_{j}$), the function *T* determines the probability of finding the gas source after executing the action *a*.
- *S(γ)* is a tree-based representation of the problem state space when all possible actions *a* are executed for a given list of object locations, within a finite time-horizon specified by γ.
- The reward function *R* depends on the action *a* that was executed as well as on the reward of the previous state. That is, it evaluates the reward of visiting a particular object location and finding out whether it is the gas source, also considering the *cost* of the decisions taken before reaching that state. The parameters used to specify it are the *navigation and validation times* for the previous and current states, and a *bonus value* the system receives if the inspected object is the gas source.

Once the MDP representation is built, the *utility* of each state is obtained through the value iteration method, also called Bellman’s update [35]. Given a current search state *s*, the next object to visit corresponds to the reachable state *s’* with the highest utility. Once the navigation to the object represented by *s’* is completed, we check it for being the gas source. If it is, the task has finished. Otherwise, the object is removed from the list of remaining candidates, and the MDP is updated with the new information, obtaining a new target candidate.

The complexity of the MDP representation drastically increases with the number of objects, making its building intractable. To handle this, we consider the notion of *horizon*, which fixes the maximum depth of the MDP topology. This means that, for example, with *horizon = 2*, the path planning algorithm will consider scenarios where the source is found at the first or second try, with *horizon = 3* also the third try is taken into account, and so on.

Recall that we are considering the case where the gas source is not among the objects already recognized by the robot (i.e., the gas source is not contained in the list of source candidates). Due to this possible scenario, two different strategies were implemented, as described in Section 3.5, to account for new objects in the workspace.

## 4. System Evaluation

**Figure 3:**
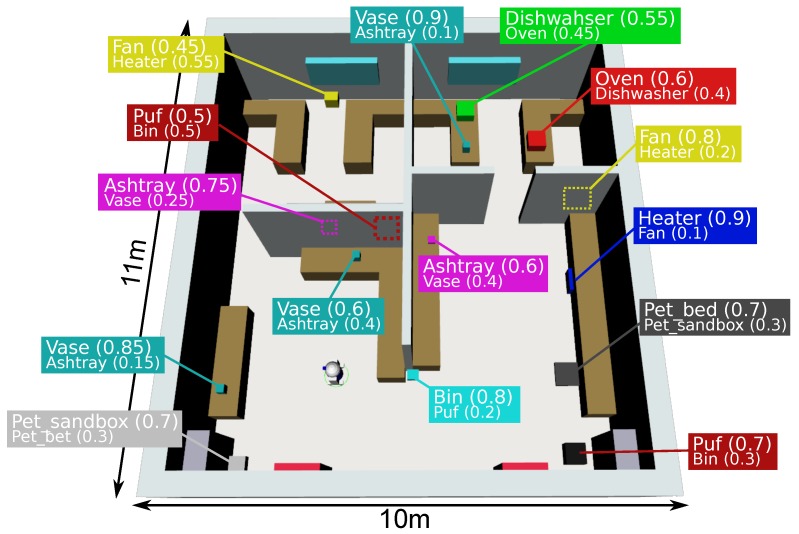
3D simulated home environment composed of four interconnected rooms and fifteen object candidates. Objects are shown as 3D colored boxes specifying their location in the environment and their categorization probabilities.

This section presents a simulated experiment where a mobile robot equipped with an e-nose must locate a gas emission source in a 3D human environment with multiple objects (see Figure 3). We considered three gas classes, Smoke_smell, Gas_smell and Rotten_food_smell; 11 object categories (Vase, Bin, Ashtray, Oven, Heater, Dishwasher, Fan, Puf, Incense_stick, Pet_sandbox and Pet_bed); and 15 different objects, which we assume have been previously detected by the robot with the probabilities shown in Figure 3.

For illustrative purposes, and since the simulated environment allow us to run several methods under identical circumstances, we compared our approach (*semantic-mdp*) with a naive approach that searches for the gas source by randomly selecting the next candidate from the list of objects in the environment (*random*). The latter can be interpreted as a reasonable baseline to compare with. Furthermore, to determine the importance of the different components in the proposed search strategy (i.e., the source probability estimation (Section 3.4) and the MDP path planning (Section 3.6)), we also compared with the cases of: (i) searching based only on distances to the candidates, selecting the closest object as the next candidate to validate (*greedy-d*); (ii) based only on the estimated source probability, that is, inspecting the different objects by decreasing source probability order (*semantic-p*); and (iii) with a more elaborated configuration from a previous work [37] that considers the cost function $L=-ln\left(P(S=o_{i}|z_{g},z_{v})\right)\cdot d_{o_{i}}$ accounting for the source probabilities and the distances from the robot location to the candidates $d_{o_{i}}$ (*semantic-cost*).

To obtain statistically representative results, we evaluated each search strategy *500 times* and report the mean and standard deviation for three evaluation measures: the overall travelled distance *d*, the number of inspected objects *n*, and the global search time $t_{s}$ (recall Equation (1)). Furthermore, to assess the expected behavior of the tested strategies under different circumstances, for each evaluation we randomized the following parameters:

- **The gas source**, randomly selecting an object to be the gas source from the list of objects in the environment.
- **The class of the released gas**, simulating a gas dispersion in accordance with the types of gases the selected source can emit.
- **The initial robot location**, selecting a random pose within the simulated environment.
- **The gas classification probabilities**, evaluating the robustness of the different search strategies for a decreasing success in the gas classification. To do so, we defined the *ambiguity factor* in [30] as $\alpha =1/P(G_{k}^{*})-1$, where $P(G_{k}^{*})$ is the classification probability assigned to the gas being released. That is, α models the ratio between the probabilities assigned to the other gas classes and the probability of the gas being released, providing values close to zero for good classifications, and high values for bad, ambiguous ones. Furthermore, we also report an average case where the classification probabilities were randomized in the range α∈[0,inf], that is, from very accurate to complete failure.

Finally, with respect to the three parameters that govern the MDP, i.e. the horizon *h*, the discount factor γ, and the bonus factor (recall Section 3.6), we carried out a cross-validation fitting to select the best combination from more than 200 different configurations. For the comparison with other search approaches shown below, we report the results of the best configuration for each ambiguity range.

Table 1 illustrates the comparison of the different search strategies for varying ambiguities in the gas classification, depicting the average distance travelled by the robot, the number of objects visited before detecting the source location, and the overall search time. For the latter. we considered an average robot speed of v=0.4 m/s and a validation time of $t_{v}=30$ s.

From analyzing these results, we can reach the following conclusions:

- The *greedy-d* approach, being independent of the object categorization and gas classification results, outperforms the *random* case, especially with respect the travelled distance. However, both approaches lead to long searches (over 5 min for the tested environment).
- Introducing the semantics framework to generate the probability ordered list of candidates notably reduces the number of visited objects, and consequently the overall search time (see *semantic-p*). This is specially noticeable when the ambiguity in the gas classification is low.
- In general, when a trade-off between source probability and navigation cost is considered, the search time reduces. An exception is the *semantic-cost* approach, which seems to yield better results only when there is high ambiguity in the gas classification (see bottom row in Table 1). This suggests that fusing source probability with navigation costs is not straightforward.
- The proposed *semantic-mdp* approach provides the best results overall, independently of the gas classification ambiguity, achieving search times below 2 min in most cases.

## 5. Real Experiment

We demonstrated the usability of our SGSL under real-world conditions by analyzing a trace of a real experiment carried out with a wheeled mobile robot in an uncontrolled human environment. Notice that, during the experiments, no actions were taken to control the environment or the dispersion of gases, that is, no artificial wind flow was forced and we did not restrict access to the search area, allowing personnel to keep their daily routines. The only alteration in the environment for the realization of this experiment was the inclusion of different objects to enrich it and rise the complexity of the search.

### 5.1. Experimental Setup

**Figure 4:**
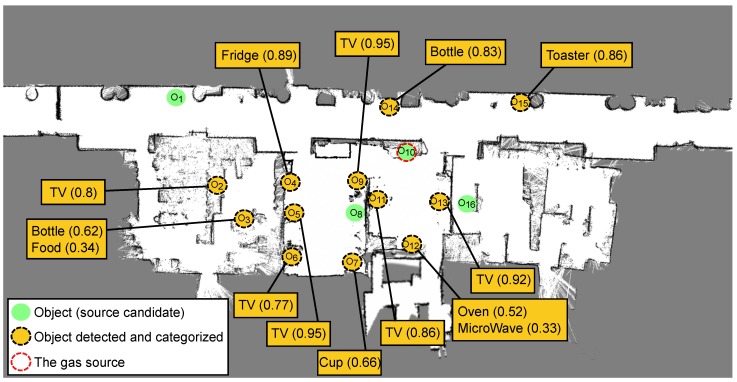
Occupancy grid map of the environment with detailed location of the objects present on it and their respective categorizations as outputted by the vision-based object recognition system. When the source search is triggered, only 12 out of 16 potential candidates have been detected, not including in this set the gas source.

Figure 4 illustrates the experimental area by plotting the occupancy map built by the robot and the location of all the objects in the environment (marked with a circle). We assumed that the robot has already inspected the environment and has detected some of these objects (marked with a solid-orange circle), but not all of them (solid-green circles represent objects in the environment not yet detected and categorized). To illustrate the dynamic nature of our SGSL approach, the gas source for this experiment (marked with a dotted-red circle) was not included in the initial set of candidates. Concretely, objects not considered at the start of the search include: $O_{1},O_{8},O_{10}$ and $O_{16}$.

**Figure 5:**
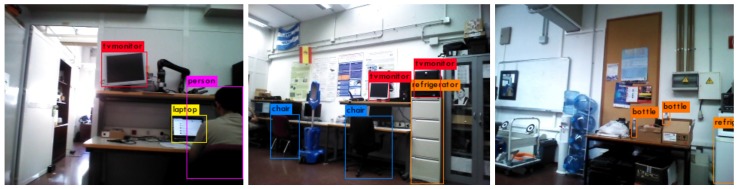
Categorization results for some of the objects considered in the real experiment. As can be noticed, the object recognition system is not exempt of errors, incorrectly categorizing objects in some occasions (e.g., see storage cupboard miss-categorized as *Fridge* in the central picture).

Figure 4 also depicts, for the detected objects, their categorization as result of the visual recognition system (see Section 3.2). For practical reasons, we only considered those categories with a belief above an empirically set threshold (0.3 for the current experiment). This leads to most object instances to be categorized as belonging to only one object-category, and few instances with two different categories (i.e., in those cases where the systems is not very sure of the categorization). To illustrate this initial categorization, Figure 5 shows some pictures taken during the process. As can be noticed, the object recognition system is not exempt of errors, sometimes incorrectly categorizing objects. Finally, to complete the information needed to perform the SGSL, Table 2 shows the conditional probabilities that relate the object categories and the gas classes considered in the experiment.

### 5.2. The Robot and the Gas Source

**Figure 6:**
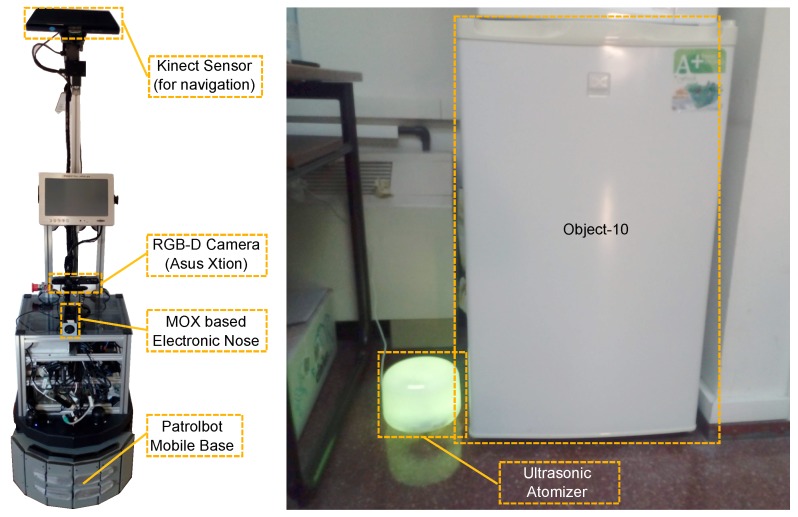
(**Left**) picture of the mobile robot employed during the real experiments. Besides the standard sensors used for navigation (2D lasers and Kinect), the robot was equipped with an additional RGB-D camera to perform object recognition, and a MOX-based e-nose [38] for gas detection and classification. (**Right**) photo of the ultrasonic atomizer releasing a mixture of water and ethanol next to the fridge (*Object-10*).

Figure 6 (left) shows a picture of the mobile robot employed in the experiments, a Patrolbot platform from Mobile-Robots equipped with two 2D range finders (Hokuyo UTM-30LX and Sick LMS-200) for localization and autonomous navigation and a Kinect sensor for collision avoidance (obstacle detection). In addition to these sensors, we installed a supplementary RGB-D camera (Asus Xtion Pro-Live) for object detection and categorization and a MOX-based e-nose (MAPIR-nose [38]) for chemical sensing. Further details about the robotic platform can be found in [39].

We considered the object releasing the gases in the environment is a fridge (*Object-10*), and that the released gas is smoke. For safety reasons. we cannot reproduce harmful leaks within the laboratory, so, for the purpose of this experiment, we matched ethanol to *smoke_smell*, acetone to *gas_smell* and floor soap to *rotten_food_smell*. Consequently, to generate the leak, we considered an ultrasonic atomizer containing a mixture of water and ethanol at 50%. Figure 6 (right) shows a picture of the ultrasonic atomizer releasing the mixture of gases next to *Object-10* location.

### 5.3. Experimental Results

**Figure 7:**
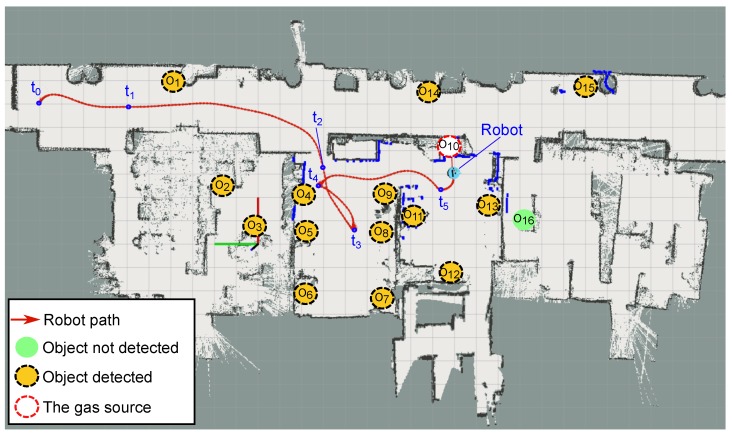
Path followed by the robot while searching for the gas leak (located at *Object-10*) and final state of the different objects considered in the experiment. Different time-instants were analyzed to account for the new discovered objects and estimated utilities.

Figure 7 displays the path followed by the robot during the execution of the experiment. It started (t=0) at the upper-left corner of the map with an incomplete list of object candidates (see Figure 4), and then visited different objects in the environment according to the information available at each time instant until the source was reached. We analyzed different time instants of the search process depicting the utility of each object in the environment and indicating the selected one to be checked by the robot.

**Figure 8:**
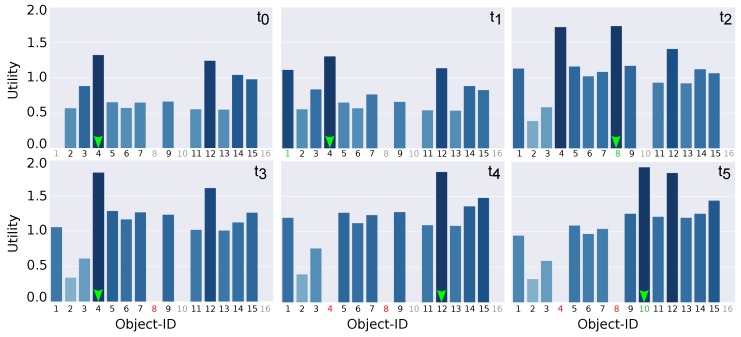
MDP utilities associated to each candidate object at the different time steps. The ID of each object is colored as grey if not yet detected, green when the object has been detected and introduced in the candidates list, and red when the object has been discarded as not being the source. The candidate with the highest utility is marked with a green arrow.

- t=0: The first task carried by the robot, once the search was triggered, was the classification of the detected gas. Using a Naive–Bayes classifier and the MOX-based e-nose mounted on the robot, the system classified the gas leak with the following probabilities: 0.56 (smoke), 0.28 (gas) and 0.16 (rotten_food). Notice that this ambiguity in the gas classification led to objects not potentially releasing smoke (e.g., an apple) to be considered as possible source candidates, naturally with less probability. Recall that, if the classification is erroneous or too ambiguous, the search process may suffer from important delays, as depicted in Table 1.
  Once the gas was classified, the system estimated the utility of each object candidate. However, in this step, only 12 out of the 16 objects in the environment were considered. This led to only 12 objects being assigned a utility by the MDP, as can be seen in Figure 8. With this limited knowledge, the system selected *Object-4* as the next candidate to check and started navigating towards it.
- t=1: A few seconds later, the vision system detected an apple (*Object-1*) and categorized it as *Food* with a belief of 0.93. This new object was automatically introduced as a source candidate and the MDP was re-run. As can be seen in Figure 8, *Object-1* was now assigned with a utility value. Despite *Food* not being a category likely to release *smoke*, the uncertainty in the gas classification together with its proximity to the current robot location led to a relatively high utility (third best candidate). However, the best candidate remaind *Object-4*, so the navigation remained unaltered.
- t=2: Once the robot left the corridor and entered one of the rooms present in the environment, it detected a new object (*Object-8*) which was categorized as *Oven* with a belief of 0.93. As in the previous case, the object was introduced as a potential candidate and the MDP was re-run. In this case, the new object was estimated to have the highest utility among the current candidates, so the robot pleace the current navigation to *Object-4* on hold, and started a new one to *Object-8*.
- t=3: Once the navigation to *Object-8* was completed, the system validated that it was not the gas source. As in most gas source localization works, we did not face a real validation stage (a generally complex process involving several measurements and assumptions about the wind flows in the environment), but assumed the robot could accurately carry out this task. Therefore, *Object-8* was removed from the list of candidates and the next object in the list with the highest utility was selected. In this case, *Object-4* was re-selected as the most promising object to check. Notice that, since the robot had changed its location, the utilities of all objects vary, being necessary to update their values.
- t=4: *Object-4* was a storage cupboard miss-categorized by the recognition system as a *Fridge*. Naturally, once the robot reached the object, it was determined that it was not the gas source. After removing *Object-4* from the list and updating all the remaining object utilities, the next selected candidate was *Object-12*.
- t=5: On its way to *Object-12*, the robot detected a new candidate (*Object-10*) and categorized it as *Fridge* with a 0.98 belief. Given the proximity to the current robot location and the fact that *Fridges* are likely to release *smoke* (see Table 2), it became the candidate with the highest utility, and a new navigation was commanded to check it (placing the navigation to *Object-12* on hold). Once the robot reached *Object-12* location, we assumed the gas source had located and the experiment concluded.

In this way, this trace illustrates how our SGSL system performs in a real environment once a smell has been detected, and how it dynamically reacts when new objects are detected during its search.

## 6. Discussion

Having described in detail the proposed method for gas source localization combining vision, chemical sensing, and semantic knowledge, and after its thorough validation under challenging conditions, in this section, we discuss the key components that play an important role in determining the overall system performance. Those components, namely the object recognition sub-system, the gas classification sub-system and the a priori expert knowledge in the form of conditional probabilities, are confronted separately to clearly state the limitations of the proposed approach.

- **Object Recognition:** The proposed SGSL system relies on a set of object candidates found in the environment to solve the GSL problem. Therefore, the detection and categorization of these objects play a fundamental role in the final performance of the system. The main issues to handle are twofold: (i) objects not detected; and (ii) objects detected but incorrectly categorized.
  The former is a critical problem since non-detected objects will never be considered as potential gas sources (independently of the gas released or the provided expert knowledge). Again, two situations may arise: an object has not yet been observed (e.g., it was introduced after the initial robot inspection, it was occluded by other objects, etc.) or the object was observed but the recognition system failed to detect it (something that highly depends on the detection method employed, being mandatory to use a well tuned method able to robustly detect the object categories relevant to the problem at hand). Our approach is robust against the former by implementing different dynamic object detection modes which allow the robot to consider new objects (recall Section 3.5). For example, if the gas source is not found after checking all the candidates in the list, a full search should be commanded to look for new objects not previously detected.
  The second issue is the miscategorization of a detected object. Regardless of the performance of the chosen visual detection sub-system, categorization errors are to be expected in real world environments and, therefore, the system should to be able to deal with them. This is the main reason for not considering deterministic categorization methods but probabilistic ones. The latter provide a probability for each object category instead of Boolean values (i.e., 0 or 1). Therefore, even when failing to determine the object category (usually the label with the highest probability), our SGSL approach will keep the object as a source candidate. The downside of assigning low probabilities to the correct object categories is an efficiency drop in the source-probability estimation phase, leading to longer inspection times. In any case, the system will eventually locate the correct gas source, though the system efficiency in terms of search time would be downgraded.
- **Gas Classification:** Similar to the object detection sub-system, a proper and accurate classification of the gas whose source is to be located largely affects the system efficiency. As illustrated in Section 4, when the gas classification fails, the overall search time increases, being necessary to visit a higher number of candidates before locating the gas source. However, as long as the probability assigned to the correct gas class is non-zero, the true gas source will eventually be located by the proposed approach.
  Finally, we need to highlight the importance of properly designing the e-nose system to react to the concentration ranges to be expected in the application. As with the detection of objects, an e-nose unable to detect the presence of a gas spread in the environment (e.g., because its minimum detection limit is higher than the gas concentration) will lead to a complete failure, not even triggering the search process. In this sense, it must be stressed that this limitation is not present only in our approach, but shared by all the methods that rely on sensor measurements to solve the GSL problem.
- **Expert Knowledge:** The proposed system contemplates expert knowledge in the form of conditional probabilities to link object categories with gas classes. In a nutshell, the system exploits knowledge about which object categories are likely to release each gas class. As any other method built upon knowledge from an expert, our approach requires this knowledge to be updated according to the application. For example, in the experiments shown above, we used the conditional probabilities depicted in Table 2 to illustrate our knowledge in the office-like scenario, but, if the system were to be used for explosive detection, then the conditional probabilities should be replaced to uniform ones along all object categories, indicating that all objects are potentially candidates to hide explosive substances. Besides, taking into consideration the limitations when materializing the expert knowledge as simple conditional probabilities, we can find situations where this semantic information is not accurate or even misleading. Once again, the only “fatal” situation in which errors in such probabilities would lead to a complete failure of our approach are those where absolute zero probabilities were assigned to some object–gas combination. Therefore, the final responsibility lies on the expert to properly assign the values to represent its knowledge about the objects and gases in the system.

Summarizing, due to the variety of fields involved in the presented approach, the overall performance of the system is affected by many factors. We have analyzed the most important ones and discussed their influence to determine when the system would fail and when a deterioration of the performance can be expected.

## 7. Conclusions

In this paper, we have revised the problem of gas source localization with a mobile robot within a real-world human environment and proposed a new approach that accounts for the semantics between the detected gas and the objects in the environment to deal with this challenging problem. Our approach relies on a set of visually recognized candidate objects, an ontology that naturally encodes the relations between objects and the gases they are likely to release, and a probabilistic Bayesian framework to fuse such information in a useful way. These factors makes this approach particularly suitable for structured indoor environments containing multiple objects likely to release gases where semantic relationships can be exploited.

To leverage the estimated gas source probability of each object in the environment, and with the aim to reduce the search time, we have proposed a Markov decision process that also takes into account the cost derived of both, navigating to the different objects, and validating them (i.e., discerning if the object at hand is the gas source). As a result, our approach is able to provide an inspection plan that maximizes the utility and reduces the overall search time.

One of the strengths of our approach when deployed in real-world environments is the non-dependency of a continuous measurement of the gases dispersed (i.e., neither performing plume-tracking nor relying on consecutive gas hits). This enables our approach to work under challenging conditions where the gas dispersal is chaotic and dominated by turbulence. Furthermore, the proposal takes into account the non-negligible uncertainty coming from both the gas classification and object recognition processes. However, exploiting the high level relationships between objects and gases also brings some disadvantages, such as the strong dependency with the object recognition system. Only objects successfully detected and categorized can be considered as potential gas sources, so a proper tuning of this component is mandatory to ensure the usability of the proposed SGSL.

The suitability of our proposal has been demonstrated in both simulated and real-world scenarios, containing multiple objects and rooms, and with realistic uncertainties. A comparison between different path planning approaches has also been provided, suggesting that the consideration of semantics and uncertainty represents a promising approach for tackling this challenging problem.

As future work, we plan on comparing our approach with traditional GSL methods. This, however, requires in most cases the adaptation of the GSL methods to work under human-like environments, which is not straightforward.

## Figures and Tables

**Table 1:** Travelled distance (*d*), number of inspected objects (*n*) and average search time ($t_{s}$) for different search strategies and gas classification ambiguities (α).

α		Random	Greedy-d	Semantic-p	Semantic-Cost	Semantic-MDP
	**d (m)**	77.72±44.67	23.24±16.75	19.41±13.85	14.59±11.67	11.77±7.84
[0−0.5]	**n**	8.51±4.51	7.47±4.11	2.43±1.05	3.53±2.08	2.23±1.04
	**$t_{s}$ (s)**	449.60	282.20	121.43	142.38	96.33
	**d (m)**	81.49±45.99	23.76±16.33	22.77±16.59	16.54±12.26	14.08±9.25
[0.5−1.0]	**n**	9.10±4.62	7.73±4.13	2.89±1.39	4.09±2.34	2.57±1.31
	**$t_{s}$ (s)**	476.73	291.30	143.63	164.05	112.31
	**d (m)**	77.15±45.41	23.82±15.89	24.07±18.98	16.70±12.83	17.01±10.84
[1.0−1.5]	**n**	8.52±4.62	7.78±3.97	3.25±1.78	4.31±2.42	2.88±1.60
	**$t_{s}$ (s)**	448.48	292.95	157.68	171.05	128.92
	**d (m)**	82.54±48.02	24.96±17.06	37.50±30.54	17.53±12.82	14.69±11.04
[0−inf]	**n**	8.84±4.60	7.90±4.12	4.11±2.60	4.49±2.43	2.75±1.77
	**$t_{s}$ (s)**	471.55	299.4	217.05	178.53	119.23

**Table 2:** Conditional source probabilities for each combination of object category and gas class: $P(S=o_{i}|C_{j},G_{k})$. As can be seen, some categories do not release any of the gas classes considered in the experiment, an aspect to be exploited by our system, together with the object recognition uncertainty, to locate the gas source.

Category	Smoke_Smell	Gas_Smell	Rotten_Food_Smell
TV monitor			
Food			0.17
Fridge	0.25		0.17
Oven	0.25	0.5	0.17
Bottle		0.5	0.17
Microwave	0.25		0.17
Cup			0.17
Toaster	0.25		
